# Antiretroviral therapy in sub-Saharan Africa: evidence about need, uptake and impact from community-based cohort studies

**DOI:** 10.1111/j.1365-3156.2011.02947.x

**Published:** 2012-07-30

**Authors:** Jim Todd, Ally Wringe, Sian Floyd, Basia Zaba

**Affiliations:** London School of Hygiene and Tropical MedicineLondon, UK

**Keywords:** community, Africa, cohort, antiretroviral therapy

Antiretroviral therapy (ART) has transformed the lives of individuals with HIV in many parts of sub-Saharan Africa (UNAIDS 2011). In common with other regions of the world, after starting ART, people living with HIV have survived longer, regained their health, and resumed their economic activities. However, with more than 20 million people infected with HIV on the African continent, helping only a proportion of them is not enough. We need to know how ART is affecting the HIV epidemic at the population level: how many HIV positive people need ART according to current WHO guidelines, how many are taking ART and how many lives have been saved. National statistics on the numbers of people receiving ART or on the number of drugs delivered do not tell us whether access is equitable or what the overall impact is on the population.

The data used in this supplement to *Tropical Medicine and International Health* come from population-based cohorts where HIV status has been measured over many years and from the analysis of demographic and health surveys (DHS). Longitudinal cohort studies that include data on HIV infection are a valuable resource to help us understand the impact of the epidemic in the community (Maher *et al.* 2010). They also enable us to see, at population-level, how well we are doing in our efforts to care for and treat HIV-infected people and their families. This collection of papers provides important evidence on the ways we can improve access to HIV Counselling and Testing (HCT) and uptake of ART. This will add to the information that has already been assembled by the ALPHA network on the prevalence and incidence of HIV in these communities, as well as on individual-level mortality and the impact of HIV on families living in these communities (Hosegood *et al.* 2007; Todd *et al.* 2007).

As the first step, Zaba *et al.* (2012) present a new method for estimating the need for ART. Based on earlier studies by the ALPHA network sites on mortality among HIV-infected individuals, this approach simplifies the calculations for estimating the population-level need for ART. The method can be applied to any population in which the age-specific prevalence of HIV is known. While it does not allow the identification of individuals in need of ART, it does allow national, district and community level planners to predict the overall need for ART over several years. This method has many advantages, but before it can be used more widely, it needs to be applied in cohort studies to establish its credibility.

Uptake of HCT is the next step considered. The paper by Isingo *et al.* (2012) analyses trends in HCT in Northern Tanzania, where HIV surveillance in combination with mobile clinics offering HCT has boosted the numbers who know their HIV status. Cremin *et al.* (2012) analysed DHS data on trends in self-reported testing for HIV, which include all types of HIV testing. These papers show gradual increases in the proportion who have ever tested for HIV over time, with the highest proportion in those with more education, and in those who are HIV positive; this probably reflects knowledge of ART and its impact on those living with HIV. The paper by Wringe *et al.* (2012) shows how different testing strategies for HIV affect the numbers that know their status. This has implications for HIV testing programmes that are trying to ensure that everyone in the population knows their HIV status.

Uptake of ART is the important step that transforms the lives of those living with HIV. Two papers, from Tanzania and Uganda, describe the factors that are associated with uptake of ART in the population. Wringe *et al.* (2012) show how the numbers who obtain ART build up slowly when the service is only available in a referral hospital many miles away. In contrast, Kazooba *et al.* (2012) reveal the benefits of a single local clinic that can take patients through all the steps from testing and counselling to the provision of ART, with more than 66% of those in need of ART receiving it in 2008.

The impact of ART can be measured in several ways, but one of the most important is the effect on all-cause mortality. With the rising uptake of ART since public-sector provision began in 2004, and longer survival of those starting and remaining on ART, four papers show its population-level impact on mortality in the community. We present evidence from Tanzania, Uganda and Malawi (Marston *et al.* 2012; Kasamba *et al.* 2012; Chihana *et al.* 2012). Floyd *et al.* (2012) compile the evidence from these three settings with data from the Africa Centre DSS in South Africa to show large population-level reductions in mortality when ART provision has been localised and/or decentralised. This reinforces the anecdotal evidence from these communities, where carpenters no longer only make coffins for the dying, but can resume making furniture for the living. The impact of ART can also be seen in the incidence of orphanhood in the communities, as shown in the paper by Makumbi *et al.* (2012), which reminds us that survival of a parent has wide implications for the whole family.

The final paper in the supplement, by Bernighausen *et al.* (2012), points at an important new direction that community surveillance studies should be taking. By linking community data with data from clinics, it is possible to get a more accurate picture of the uptake and continuity of service use, not only HCT and ART but also family planning, antenatal clinics and other treatment services. This article shows that new technology and software can be used to enable these links.

The overall message from these papers is that we have progressed a long way since antiretroviral therapy first became available in sub-Saharan countries in 2004. However, major discrepancies remain in access to, and uptake of, ART. Obtaining reliable data on access, uptake and the population-level impact of ART is difficult, and community-based studies such as those that have contributed to this supplement, are a very valuable resource. Going forward, we will need to use new technologies to get as full as possible a picture of how to ensure that these services reach the people who need them.
